# 

**Published:** 1877-12

**Authors:** 


*CONTENTS.*

| | PAGE |
|---|---|
| OUR QUARTER CENTENNIAL YEAR | 323 |
| TO STOP A COUGH | 324 |
| CANCER CURED BY ELECTRICITY | 326 |
| SHAVING THE BEARD | 329 |
| BUCKWHEAT CAKES | 329 |
| NATURE'S METHODS | 330 |
| THE HUMAN TEETH | 332 |
| TEXAS WOOL, OIL, AND BEEF | 333 |
| FLOWER POTS | 334 |
| VENTILATION | 335 |
| BRAIN WORKERS | 337 |
| TUBE WELLS | 339 |
| PERFECT MILK | 340 |
| HOT AIR FURNACES | 341 |
| WARTS | 342 |
| HUNGER | 342 |
| DR. HALL'S PORTABLE SPIROMETER | 343 |
| BATHING | 344 |
| CONSTIPATION | 345 |
| BRISTOL'S AND EDWARD'S SHOES | 346 |
| COUGH REMEDIES | 347 |
| NEW BOOKS | 347 |
| BOWEL COMPLAINTS OF CHILDREN | 348 |

